# Synthesis and Antimicrobial Specificities of Halogenated Tryptophan-Containing Nisin Variants

**DOI:** 10.1021/acschembio.5c00632

**Published:** 2025-09-12

**Authors:** Chenhui Wang, Sanne Tervoort, Oscar P. Kuipers, Jaap Broos

**Affiliations:** Department of Molecular Genetics, Groningen Biomolecular Sciences and Biotechnology Institute, 3647 University of Groningen, Groningen 9747AG, The Netherlands

## Abstract

Antimicrobial peptides, and in particular ribosomally produced and post-translationally modified peptides (RiPPs), are a potentially important class of candidate antibiotics for combating multidrug-resistant bacteria. Introduction of a halogenated Trp residue into a RiPP can possibly enhance antimicrobial efficacy and alter specificity, but this modification has hardly been explored. This study employs an efficient expression system utilizing a tryptophan auxotrophic *Lactococcus lactis* strain to biosynthetically and efficiently incorporate halogenated tryptophan analogues, namely 5-fluoro-tryptophan (5FW), 5-chloro-tryptophan (5CW), 5-bromo-tryptophan (5BW), as well as 5-methyl-tryptophan (5MW) at position 1 of I1W nisin A. Wild-type nisin and Trp-containing I1W nisin show a high and broad activity against four tested pathogens. However, the activity spectrum of the three different halogen atom containing nisin variants became more strain specific, as both increased and decreased activities were measured against the four tested pathogens. No trend between the chemical properties of the halogen atom (e.g., electronegativity, size) and the bioactivity of the nisin variants toward each of the four pathogens could be detected, suggesting strain specific antimicrobial activity mechanisms. These findings demonstrate that halogenated tryptophan analogues can be successfully incorporated into a bioactive RiPP produced by an auxotrophic *L. lactis* strain and underscore the utility of peptide halogenation for discovering novel antimicrobial agents with tailored pathogen specificity.

## Graphical abstract

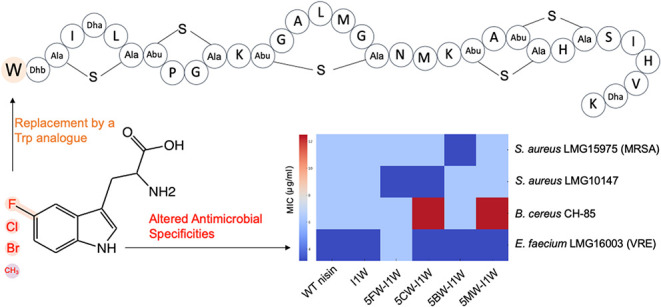

## 1 Introduction

Ribosomally synthesized and post-translationally modified peptides (RiPPs) constitute a prominent class of natural products, celebrated for their remarkable structural diversity and potent biological activities.^1^ Among the various subclasses of RiPPs, lanthipeptides are the most extensively studied. These peptides are characterized by unique thioether cross-links, including lanthionine (Lan) and methyllanthionine (MeLan), which contribute to their stability and bioactivity.^2^ Beyond these remarkable modifications, some RiPPs exhibit additional post-translational modifications, such as halogenation, lipidation, methylation, epimerization, and hydroxylation, which further diversify their structures and functional properties.^1,2^ Halogenation, in particular, has garnered significant attention due to its ability to modulate the physicochemical properties and biological activity of peptides.^3–5^ Enzymatic halogenation is typically catalyzed by FADH_2_-dependent halogenases or nonheme iron-dependent halogenases, introducing halogen atoms (e.g., chlorine and bromine) into aromatic or aliphatic substrates.^6^ For example, the lanthipeptide NAI-107, which exhibits very high antimicrobial activity, harbors a 5-chlorotryptophan (5CW) catalyzed by an FADH_2_-dependent halogenase.^7–9^ Similarly, in sponge-derived RiPPs, bromination of tryptophan at the 6-position is catalyzed by SrpI, demonstrating the diverse substrate specificity of halogenases.^10^

Halogenation has been shown to significantly impact the biological activity of secondary metabolites.^11^ For instance, the removal or substitution of halogen atoms in nonribosomal peptide antibiotics, such as chloramphenicol and vancomycin, profoundly affects their potency.^12,13^ These findings underscore the importance of halogenation as a strategy to optimize bioactive compounds. Given the precision of enzymatic halogenation, achieving comparable selectivity through synthetic methods remains challenging. Over recent years, the biosynthetic incorporation of halogenated tryptophan (Trp) analogues into peptides has emerged as a promising approach to explore the role of halogens in modulating peptide activity. In *Lactococcus lactis*, a Trp auxotrophic strain has enabled the integration of fluorinated Trp analogues (mono-, di-, tri-, and tetrafluoro-Trp) as well as monochlorinated and monobrominated Trp analogues into proteins.^14,15^ Similarly, orthogonal Trp synthetase/tRNA pairs in *Escherichia coli* have facilitated the incorporation of 5-chloro-tryptophan (5CW) and 5-bromo-tryptophan (5BW) into proteins using stop-codon suppression strategies.^16^

Despite these advances, the incorporation of halogenated Trp analogues into RiPPs remains underexplored due to challenges such as low production yields of bioengineered variants and difficulties in purification from nonstandard microbial strains. To address these challenges, our group previously explored the incorporation of halogenated Trp analogues into the well-characterized RiPP nisin, using the genetically tractable host *L. lactis*. While native nisin lacks Trp residues, single-point mutations allowed the introduction of Trp analogues at specific positions.^17^ For example, 5-fluoro-tryptophan (5FW) was successfully incorporated into nisin variants at positions 1, 4, 17 and 32, but low production yields restricted the antimicrobial activity evaluation.^17^ Recently, Guo et al. overcame this limitation by optimizing the expression system, enabling the incorporation of 5BW and 6BW into nisin variants at position 1 thus facilitating activity assays against clinically relevant pathogens.^18^

Targeting the first residue position in nisin is relevant because the N-terminal ring A- and ring B-containing part of nisin is crucial for the interaction with its target molecule, the cell wall precursor lipid II.^19,20^ Nisin mutant I1W, in which isoleucine at position 1 is replaced by Trp (Figure 1) has significant antimicrobial activity.^17,21,22^ In this study, we systematically investigated the effects of halogenated Trp analogues on the antimicrobial activity of I1W nisin. Specifically, Trp analogues substituted with a halogen atom (fluorine, chlorine, and bromine) at the 5-position on the indole ring were biosynthetically incorporated in I1W nisin, and their effects on antimicrobial activity were evaluated. By introducing different halogen atoms, a key physicochemical property is significantly altered, namely the electronegativity, with fluorine being the most electronegative element in the periodic table. Introduction of a halogen atom at the indole moiety will decrease its electron density and in turn can weaken noncovalent interactions like π–π and cation π interactions, between Trp and a nearby aromatic group or cation.^15,23^ These noncovalent interactions may play a role in the antimicrobial activity mechanism of nisin. Besides electronegativity, also atom size and polarizability of the three halogen atoms differs markedly.

**Figure 1:**
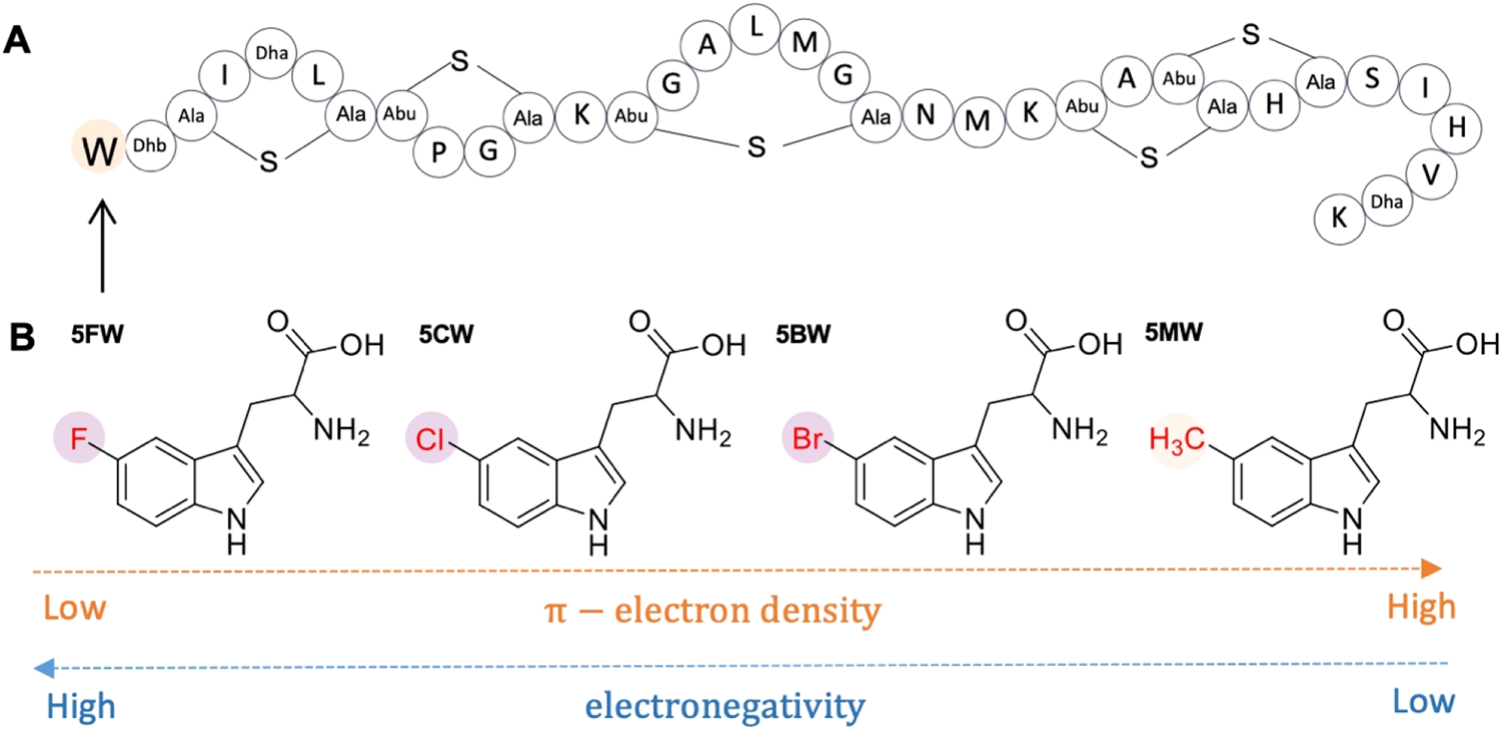
Introduction of halogenated tryptophan analogues in nisin at position 1. (A) Structure of nisin A variant I1W in which the first isoleucine is replaced by tryptophan. Dha refers to dehydroalanine; Dhb refers to dehydrobutyrine; Abu is aminobutyric acid; Ala-S-Ala is lanthionine; and Abu-S-Ala is methyllanthionine. (B) Structures of Trp analogues used in this study: 5FW, 5CW, 5BW, and 5MW.

5-Methyl-Trp (5MW) was included as a reference to take into account introducing a bulky substituent at the 5 position of Trp. Moreover, the electron density of 5MW is slightly higher than in Trp, contrasting the electron withdrawing effect of halogen substitution.^15^ This systematic analysis sought to demonstrate the feasibility of efficiently incorporating a set of halogenated tryptophans and to elucidate the relationship between halogen substitution at residue position 1 and the antimicrobial activity of nisin variant I1W, offering valuable insights for the rational design of novel antimicrobial agents.

## 2 Materials and Methods

### 2.1 General Experimental Procedures

All reagents utilized in molecular biology experiments, unless specified otherwise, were obtained from Thermo Fisher Scientific (Waltham, MA, USA). Other chemicals used in this study were sourced from Sigma-Aldrich (St. Louis, MO, USA). Specifically, 5-fluoro-dl-tryptophan and 5-bromo-dl-tryptophan were obtained from Sigma-Aldrich. 5-methyl-l-tryptophan and 5-chloro-dl-tryptophan were supplied by Biosynth Carbosynth, (Lelystad, The Netherlands).

### 2.2 Bacterial Strains, Plasmids and Growth Conditions

Details of the bacterial strains and plasmids employed in this study can be found in Table S1. *L. lactis* strains were cultured at 30 °C in GM17 medium, consisting of M17 broth enriched with 0.5% glucose. When necessary, the medium was supplemented with chloramphenicol and/or erythromycin at a final concentration of 5 μg/mL each. Peptide expression experiments were conducted in *L. lactis* PA1002. Peptide expression and the incorporation of tryptophan analogues were carried out in a chemically defined medium specifically formulated without Trp (CDM-P).^24^

### 2.3 Expression of Trp-Analogues-Containing Nisin

To investigate the incorporation of tryptophan analogues, small-scale expression and purification experiments were conducted using a 40 mL culture volume. The expression procedure was conducted with reference to previous studies.^18,24^ In the pNZ-nisin expression system, *L. lactis* PA1002 cells containing the *nisBTC* plasmid were transformed via electroporation with 100 ng of a plasmid encoding the I1W mutated *nisA* gene. The transformed cells were plated on GM17 agar containing 5 μg/mL of both chloramphenicol and erythromycin and incubated overnight at 30 °C. A single colony was selected and cultured in 4 mL of GM17 medium supplemented with chloramphenicol and erythromycin until the OD_600_ reached approximately 0.4. To activate zinc-promoter-controlled nisin modification machinery encoded by *nisBTC*, 0.5 mM ZnSO_4_ was added to the culture, and the cells were incubated for an additional 3 h. The cells were subsequently washed three times with phosphate-buffered saline (PBS, pH 7.2) and resuspended in 40 mL of a CDM-P without Trp. Following a 1 h starvation period to deplete residual tryptophan, either 250 mg/L of tryptophan or a tryptophan analogue and 10 ng/mL of nisin were added to the medium to induce nisin promoter-controlled peptide expression. After overnight incubation, the culture supernatant was collected by centrifugation at 8000*g* for 15 min and processed further for purification.

### 2.4 C18 Purification and RP-HPLC

The supernatant obtained was subjected to purification utilizing open C18 chromatography columns. These columns, containing 1 mL of C18 resin (Waters), were prepared by an initial prewash with 100% acetonitrile (MeCN) (VWR) supplemented with 0.1% trifluoroacetic acid (TFA). Following this, they were equilibrated using 100% Milli-Q water containing 0.1% TFA. The samples were subsequently loaded onto the columns, allowing the peptides of interest to bind. The columns were washed in two sequential steps: first with 100% Milli-Q water containing 0.1% TFA, and then with a solution comprising 20% MeCN and 0.1% TFA. Finally, elution was performed using 4 mL aliquots of 40% MeCN with 0.1% TFA, and the eluates were freeze-dried for further use. Trp analogue incorporation efficiencies were calculated using the LC-MS peak intensities of Trp analogue labeled- and Trp-containing precursor peptides.

To achieve enhanced purification of the core peptides, the expression cultures were scaled to 1 L per sample. Following open C18 chromatography and freeze-drying of the eluted fractions, the freeze-dried material was reconstituted in 2 mL of Milli-Q water. This solution was treated with 200 μL of the engineered leader peptidase NisP (final concentration approximately 0.17 μM) and incubated at 37 °C for 6 h to facilitate enzymatic cleavage.^25^ NisP was purified as previously described.^25^ Following cleavage, the mixture was then passed through a 0.2 μm filter and initially purified using open C18 columns. The columns were washed sequentially with 100% Milli-Q water containing 0.1% TFA, followed by 25% MeCN with 0.1% TFA. Elution was carried out using 30 mL of 40% MeCN containing 0.1% TFA, and the eluates were subsequently freeze-dried. Further purification of the core peptides was performed by high-performance liquid chromatography (HPLC) using a 1260 Infinity HPLC system (Agilent) equipped with an Aeris 3.6 μm peptide XB-C18 column (Phenomenex). The separation of peptides was performed using a mobile phase of MeCN containing 0.1% TFA, in the following gradient: 20–25% MeCN (0–10 min), 25%–40% MeCN (10–40 min), 40%–80% MeCN (40–45 min), and 80% MeCN (45–50 min), at a flow rate of 1 mL/min. The collected peptide fractions were freeze-dried and used for subsequent LC-MS/MS and antimicrobial activity assays. The purity of core peptides was confirmed by HPLC using the same chromatographic conditions.

### 2.5 Tricine-SDS-PAGE Analysis

The freeze-dried precursor peptides were analyzed by Tricine-SDS-PAGE as previously described.^26^ 10 μL of each sample mixed with 10 μL 2× loading dye was heated at 95 °C for 10 min and then loaded on the gel (4% for stacking and 16% for separating). After running, the gel was stained with Coomassie Brilliant Blue G-250 buffer for about 1 h, followed by destaining in the destaining buffer (10% acetic acid, 50% methanol) for about 2 h.

### 2.6 Liquid Chromatography–Tandem Mass Spectrometry (LC–MS/MS) Analysis

LC-MS/MS analysis was performed using a Shimadzu LC-20 XR series liquid chromatography system coupled to a Thermo Scientific Q Exactive Plus mass spectrometer operating in positive ion mode. Separation was achieved on an Agilent Pursuit XR2 C8 column (3.0 μm, 50 mm × 2.0 mm) maintained at 35 °C. The mobile phase consisted of 0.1% formic acid in Milli-Q water and 0.1% formic acid in MeCN, with a total flow rate of 0.3 mL/min. A gradient elution was applied, starting at 2% MeCN, increasing to 45% at 8 min, and continuing until a total runtime of 14 min.

The mass spectrometer was operated in positive ion mode, with MS1 resolution set to 70,000 and MS2 resolution set to 17,500. The scan range was 200–2500 *m*/*z*, and data acquisition was performed in profile mode.

### 2.7 Minimal Inhibitory Concentration (MIC) Test

The MIC of modified peptides was determined using the standard doubling-dilution technique with cation-adjusted Mueller–Hinton broth (MHB).^27^ Overnight bacterial cultures were adjusted to ∼5 × 10^5^ CFU/mL, and serial dilutions of the bacteriocins were prepared in MHB across 7 wells, covering concentrations from 0.2 μg/mL to 12.5 μg/mL. Controls included a positive control with a bacterial inoculum in MHB without bacteriocin and a negative control with MHB only. The plates were incubated at 37 °C for 18 h. The MIC was defined as the lowest bacteriocin concentration preventing visible bacterial growth. Experiments were performed in duplicate or quadruplicate.

## 3 Results and Discussion

### 3.1 Expression of Nisin Variants That Contain Halogenated and Methylated Trp Analogues

In this study, the nisin mutant I1W was labeled with three different halogenated Trp analogues, as well as a methylated Trp (Figure 1). An optimized expression system was used to incorporate Trp analogues into nisin, derived from the two plasmid pILBTC/pNZ-nisin system.^18,28^ In this system, the nisin modification enzymes and transporter (encoded by *nisBTC*), and second the nisin precursor (encoded by *nisA*), were encoded on two separate plasmids: pTLReBTC and pNZ-nisin­(I1W)-TrpRS under the control of a zinc and nisin promoter, respectively, to allow optimal modification. First, *L. lactis* PA1002 cells harboring these two plasmids were grown in 40 mL GM17 medium until the OD_600_ reached ∼0.5, at which point Zn^2+^ was added to induce *nisBTC* expression. After 3 h, the medium was replaced with a CDM-P medium (lacking Trp), and the cells were starved at 30 °C for 1 h. Next, 2 mM of Trp, 5FW, 5CW, 5BW, or 5MW, respectively, was added to the cultures, followed by nisin induction overnight. The supernatants were collected by centrifugation, purified using the open C18 columns and further lyophilized.

The samples were reconstituted in 200 μL of Milli-Q water. A 2.5 μL aliquot of the reconstituted sample was analyzed via Tricine SDS-PAGE to investigate the production level of these incorporated peptides (Figure 2). While all precursor peptides were successfully produced, their yields varied depending on the analogue. The 5MW-I1W nisin sample exhibited the weakest band, indicating the lowest production level. In contrast, 5FW-I1W nisin and 5BW-I1W nisin samples displayed yields comparable to or slightly higher than the canonical Trp-containing peptide. The yield of the 5CW sample was approximately 2-fold lower than the canonical Trp-containing I1W nisin. Tricine SDS-PAGE provided preliminary evidence supporting the use of the expression system. Taken together, the used *L. lactis* expression system yields sufficient quantities of I1W nisin for downstream structural characterization and activity evaluation.

**Figure 2:**
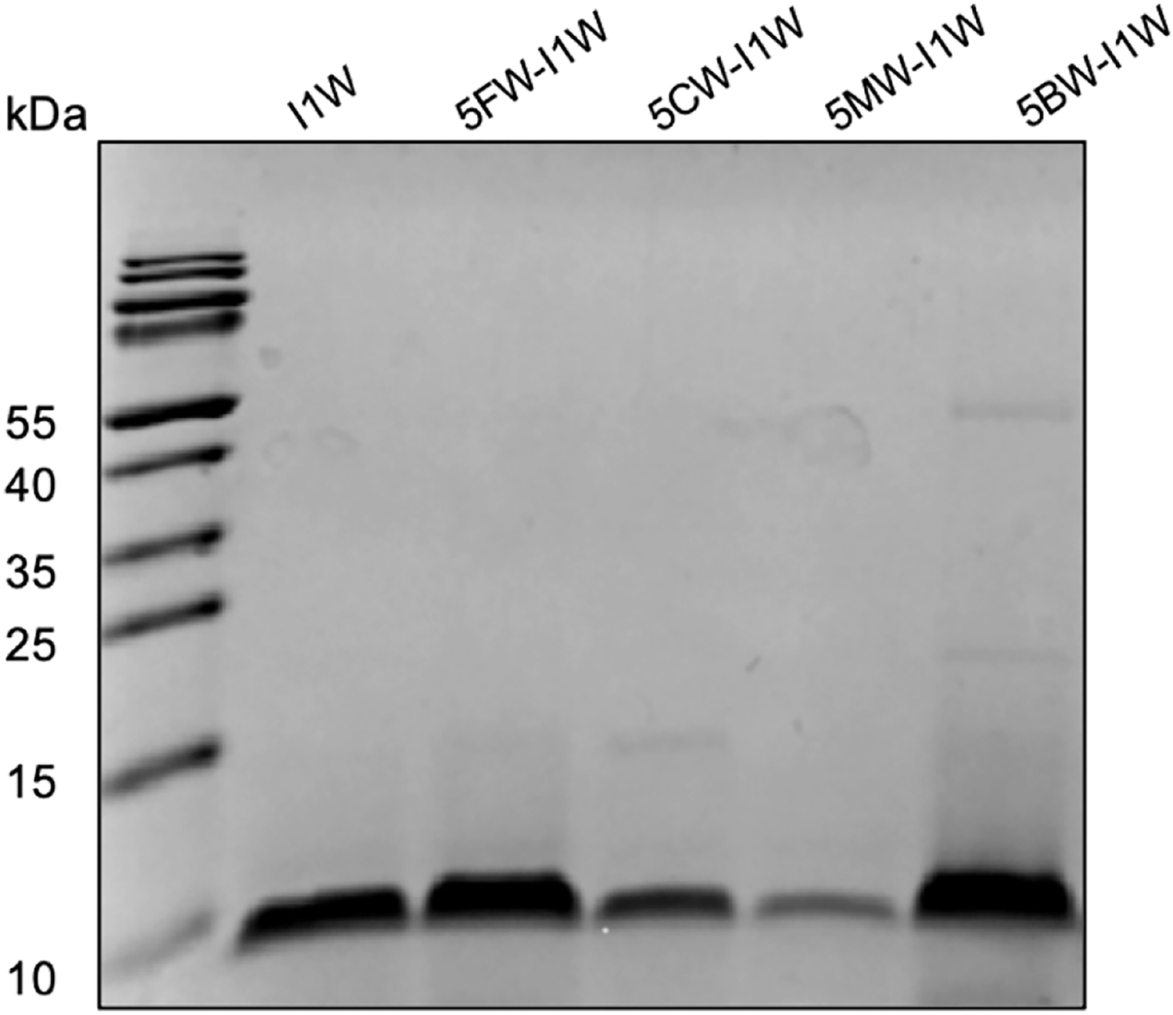
Tricine-SDS-PAGE gel, stained with Coomassie Blue, shows the production of I1W nisin expressed in the presence of Trp or a Trp analogue using a cross-expression system. Each lane contains the amount of protein purified from 1 mL of expression culture.

#### 3.1.1 LC-MS Analysis of Trp Analogue Incorporation and Dehydration Patterns

The precursor peptides were further analyzed using LC-MS to confirm the incorporation of Trp analogues (Figure 3 and Table S2). In the I1W nisin sample, peptides with eight dehydration events were predominant, corresponding with full dehydration of the I1W peptide. Smaller proportions of peptides with seven and six dehydration events, as has been shown before for other nisin variant productions, were also detected.^29,30^ Compared to the canonical Trp-containing I1W nisin, the Trp analogue-incorporated variants exhibited specific mass shifts in the LC-MS spectra and the spectra presented in Figure 3 indicate all used Trp analogues could be efficiently incorporated (86–98%) into the I1W peptide (Table S2). Notably, 5CW was for the first time biosynthetically incorporated into a peptide that belongs to the RiPPs class. Across all analogue-containing peptides, the dehydration modification pattern was consistent, with eight dehydration events predominating. However, complete dehydration was not always achieved. For example, the introduction of 5CW and 5BW appeared to impact dehydration efficiency. Approximately 65% of the peptides in these samples exhibited the full 8-fold dehydration, in contrast to the I1W mutant with canonical Trp, where more than 85% of the peptides exhibited full dehydration.

**Figure 3:**
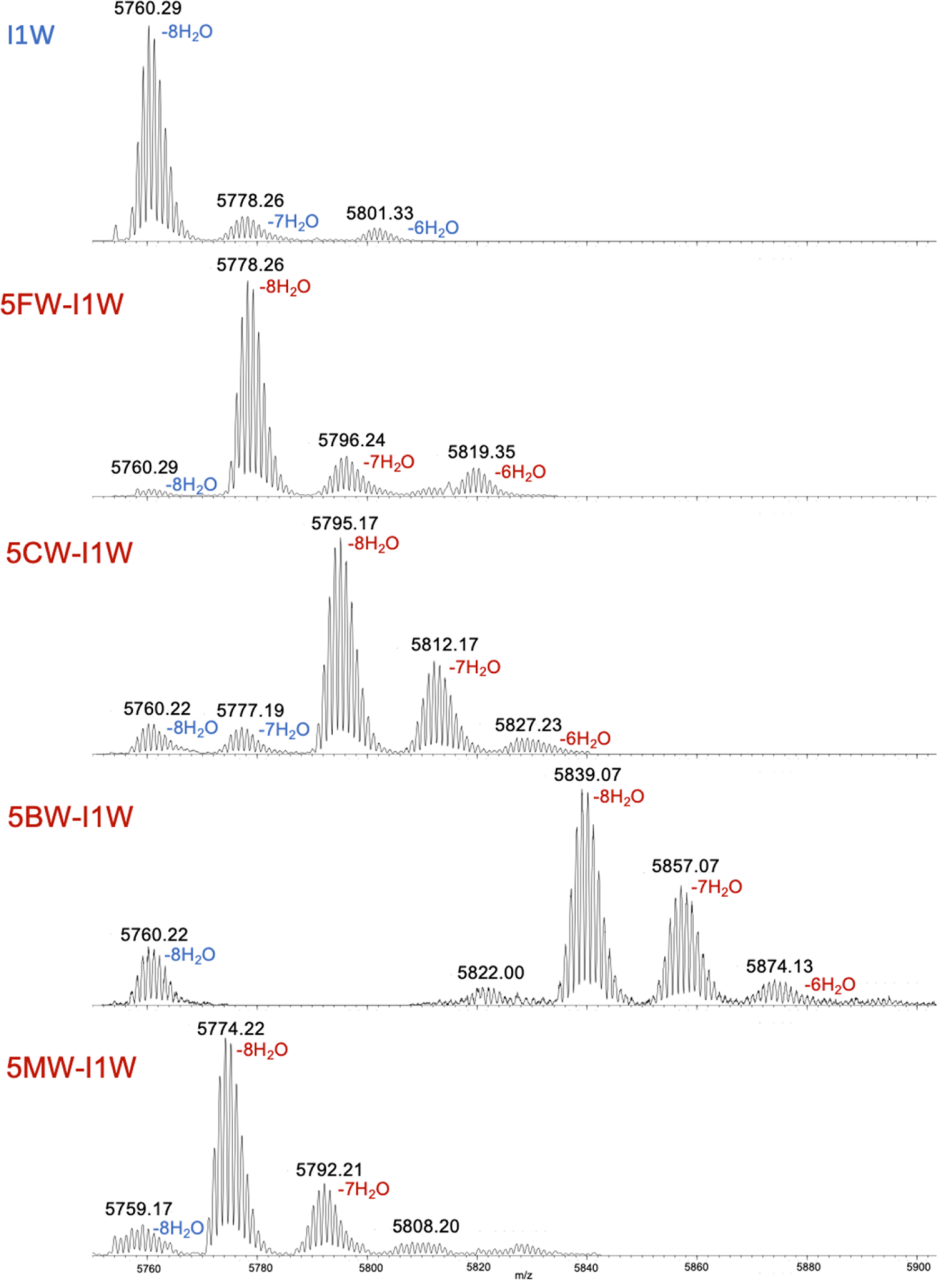
LC-MS analysis of Trp analogue incorporation into the I1W precursor peptide. The masses of peptides with incorporated Trp or Trp analogues, exhibiting varying levels of dehydration, were detected. Mass peaks corresponding to the natural Trp-containing I1W peptides (with dehydration labeled in blue) are indicated, alongside mass peaks representing Trp analogue-incorporated variants (with dehydration labeled in red).

### 3.2 Evaluation of the Ring Formation in the Core Peptides

To investigate the structural characteristics of variant core peptides and to isolate fully dehydrated core peptide products for antibacterial activity assays, precursor peptides were incubated with protease NisP to remove the leader peptide. The resulting core peptides were subsequently separated and purified using HPLC. Further structural characterization was conducted through LC-MS and tandem mass spectrometry (Figure 4). LC-MS analysis of the purified core peptides revealed fully dehydrated variants, each showing eight dehydration events (Figure 4). In particular, analysis of the quadruply-charged peaks identified an observed mass of 857.1413 Da (calculated 857.1424 Da) for the I1W peptide. The incorporation of distinct analogues resulted in specific mass shifts, including 861.6385 Da (calculated as 861.6399 Da) for 5FW-I1W, 865.6315 Da (calculated as 865.6329 Da) for 5CW-I1W, 876.6191 Da (calculated as 876.6210 Da) for 5BW-I1W, and 860.6458 Da (calculated as 860.6464 Da) for 5MW-I1W, all of which were consistent with the predicted masses of the released modified core peptides after NisP cleavage.

**Figure 4:**
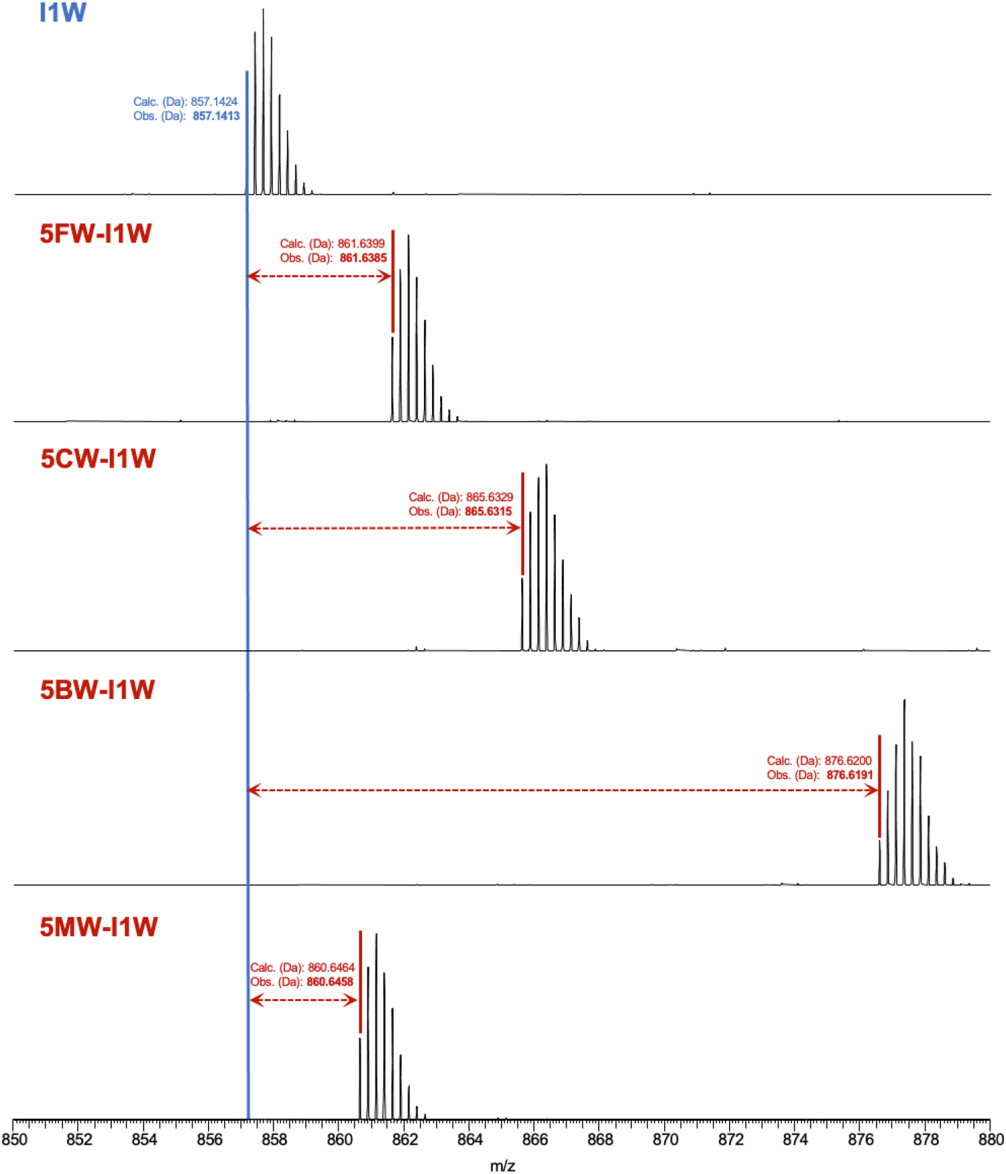
MS analysis of nisin I1W core peptides containing Trp or a Trp analogue at position 1. The mass spectra display peptide ions detected at [M–8H_2_O + 4H]^4+^, with monoisotopic masses highlighted to confirm the successful incorporation of the substituted analogues. The HPLC chromatograms of the corresponding samples are presented in Figure S2.

MS/MS fragmentation of eight-times dehydrated I1W core peptide revealed that the peptide underwent proper cyclization. Specifically, five (methyl)­lanthionine rings were formed, as evidenced by the absence of fragmentation ions within the corresponding regions (Ala3–Ala7, Abu8–Ala11, Abu13–Ala19, Abu23–Ala26, Abu25–Ala28). (Figure 5A) Similarly, MS/MS analysis of the eight-times dehydrated 5CW-I1W core peptide revealed no fragmentation within the assumed cyclized rings (Figure 5B), confirming all rings were successfully formed in the peptide structure. Additional MS/MS fragmentation analysis of the core peptides (−8H_2_O) of 5FW-I1W, 5BW-I1W, and 5MW-I1W are presented in Figure S1. The results indicate that ring formation in these core peptides is complete, with no ionized fragments detected that would suggest ring disruption.

**Figure 5:**
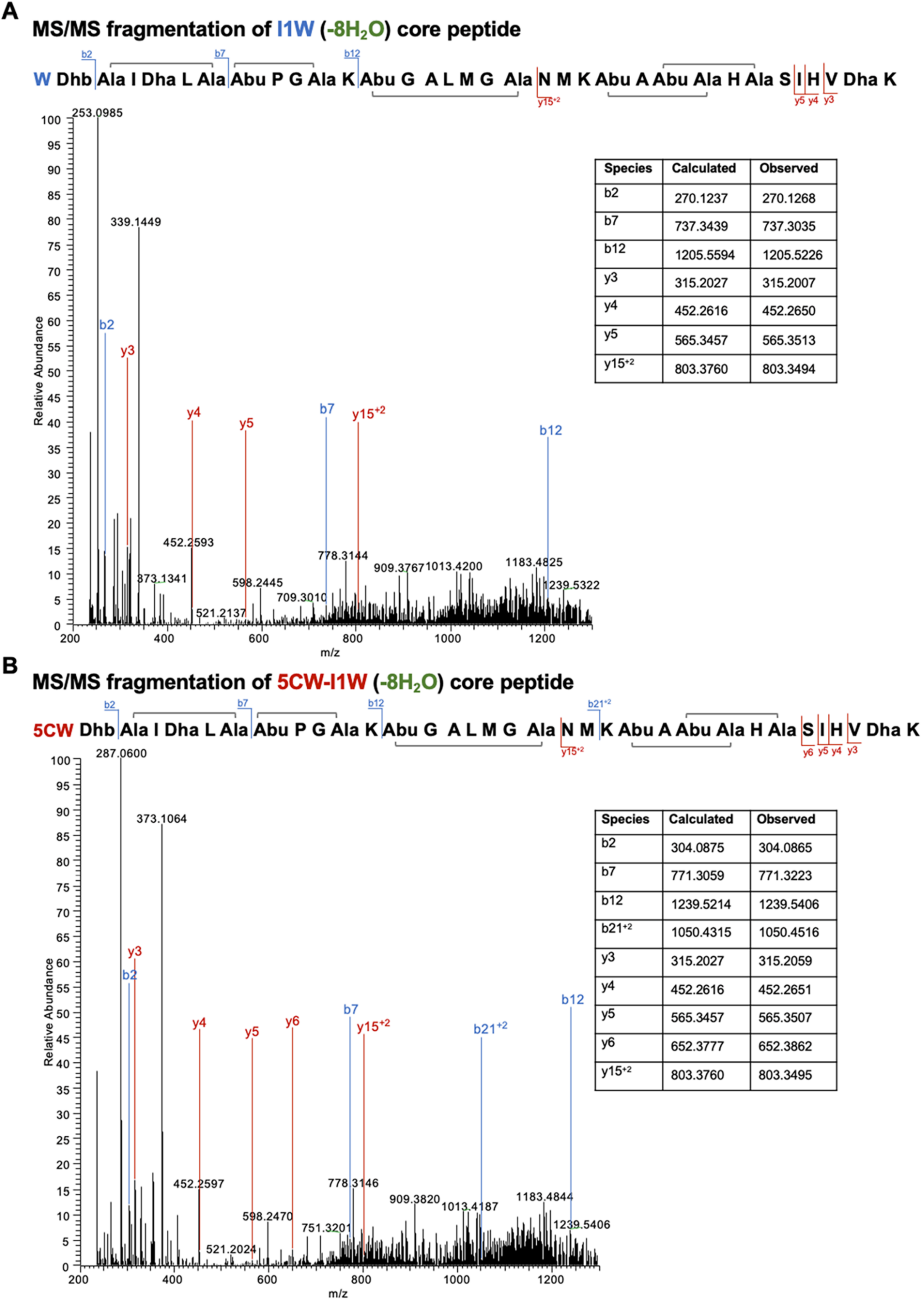
MS/MS fragmentation analysis of the I1W and I1W-5CW core peptides purified after NisP cleavage and HPLC purification. Fragmentation was derived from the [M–8H_2_O + 4H]^4+^ ion. (A) MS/MS spectrum of I1W confirms successful formation of (methyl)­lanthionine rings, as evidenced by the absence of fragmentation within the ring structures. (B) MS/MS spectrum of I1W-5CW shows a similar fragmentation pattern.

### 3.3 Antimicrobial Activity of Bioengineered Variants

The core peptide variants were isolated using HPLC and used for antibacterial activity assays. The antimicrobial activities of the variants were evaluated and compared to the natural lantibiotic nisin (Table 1). The bioengineered Trp-containing I1W variant exhibited antimicrobial activity comparable to nisin Z against the tested pathogens, indicating that replacing Ile1 with Trp had minimal impact on its overall efficacy, a result in line with a previous study.^18^ Substituting Ile1 with halogenated Trp analogues altered the antimicrobial spectrum, yielding strain-specific changes in activity.

**Table 1:**
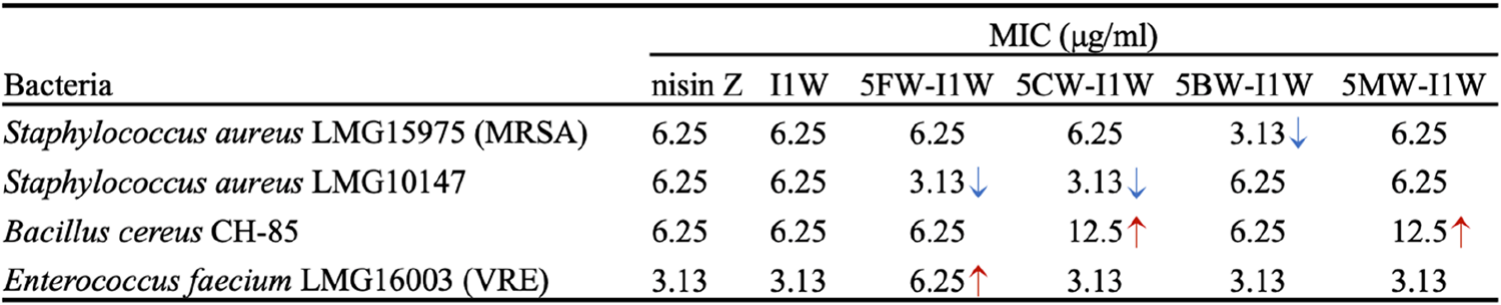
Minimal Inhibitory Concentration (MIC) of Nisin Z and Nisin I1W Variants (with Trp or a Trp Analogue Incorporated at Position 1) against Selected Gram-Positive Strains

Among the four tested pathogens, incorporation of 5FW enhanced activity against *S. aureus* LMG10147, as indicated by a 2-fold reduction in MIC value. However, this modification negatively impacted activity against vancomycin-resistant *Enterococcus faecium* LMG16003 (VRE), while activity against *S. aureus* LMG15975 and *B. cereus* CH-85 remained unchanged. Chlorination at position 1 also increased activity against *S. aureus* LMG10147. In contrast, this modification reduced activity against *B. cereus* CH-85, with no observable changes in activity against *S. aureus* LMG15975 or *E. faecium* LMG16003 (VRE). Incorporation of brominated tryptophan exhibited a distinct pattern, enhancing activity specifically against *S. aureus* LMG15975 (MRSA), while activity against the other three pathogens remained unchanged. Methylation of tryptophan failed to enhance activity against any tested strain and reduced selectivity toward *B. cereus*, indicating a generally unfavorable impact on antimicrobial performance.

Collectively, these results demonstrate that different tryptophan analogues exert distinct and target-dependent effects on antimicrobial activity. Notably, halogenation improved potency against specific *S. aureus* strains, whereas methylation led to a general loss of activity.

## Discussion

Antimicrobial peptides (AMPs) represent a significant class of candidate antibiotics to meet the urgent need to combat the increase in multidrug-resistant bacteria. Among these, RiPPs have garnered extensive scholarly and applied interest due to their diverse mechanisms of action. Halogenation is a relevant post-translational modification of RiPPs, since it has been shown to alter the antimicrobial efficacy in several cases.^18,31^ The relevance of organo-halogen compounds in antimicrobial research necessitates effective methods for the selective incorporation of halogen atoms into AMPs. Previous studies have identified the 1-position of nisin as a favorable site for the substitution of natural amino acids.^22^ This study employs a high-expression system utilizing the food-grade strain *Lactococcus lactis* to facilitate the efficient integration of Trp-5-position-halogenated analogues, specifically including fluorinated (F), chlorinated (Cl), brominated (Br), and methylated variants at the peptide’s position 1. The production yields of peptide variants differ, with the replacement of F and Br halogens maintaining expression levels similar to the wild type, while Cl halogen and the methyl group reduce expression, the latter most significantly. LC-MS and MS/MS fragmentation analyses of the core peptides suggest that the NisBC modification system is capable of accommodating various tryptophan analogues at position 1, as evidenced by the formation of fully dehydrated variants with intact ring structures. Importantly, the results clearly demonstrate that halogenated tryptophans can be successfully incorporated at position 1 of nisin and that these nisin variants with halogenated Trp analogues exhibit differential antimicrobial specificity against various pathogens. Notably, halogenation at the 5-position of Trp1 led to divergent effects against the two *S. aureus* strains tested, as well as against *E. faecium* and *Bacillus cereus* (Table 1). Specifically, the fact that 5FW and 5CW incorporation enhanced activity against *S. aureus* LMG10147, while 5BW incorporation improved activity against *S. aureus* LMG15975 (MRSA), illustrates how subtle differences in halogen atom properties can drive selective antibacterial responses. Among the four tested pathogens, the antimicrobial activities of the I1W nisin variants do not align with the order of electronegativity, suggesting a complex interplay of factors. These could include steric effects, halogen bonding, self-assembly and conformational changes.^4,32–35^ Introducing a halogen atom at the 5 position of Trp1 in nisin may alter lipid II binding or membrane interactions with some pathogens. A supporting fact is that the change in the antimicrobial activity of NAI-107 is associated with the halogenated residue located within the nisin-like lipid II binding motif.^31,36^ These findings underscore the potential of the employed methodology for peptide halogenation, thereby facilitating the development of antimicrobial products that incorporate halogenated Trp analogues with targeted efficacy against a diverse range of pathogens.

This study represents the first systematic investigation of how different halogenated Trp analogues incorporated into nisin variants affect their production and antimicrobial activity. The MIC results indicate that changes in antimicrobial activity do not correlate directly with the electronegativity, atom size or polarizability of the halogen atoms introduced. Instead, the effects of halogenation on antimicrobial efficacy vary across bacterial species, suggesting a target-specific preference for different halogen modifications. Given that the modified region lies within the lipid II-binding domain, these findings provide valuable insights into the potential for future engineering of antimicrobial peptides targeting lipid II.

## Conclusions

Collectively, this study highlights the potential of targeted halogenation to change the antimicrobial activity of lantibiotics against specific bacterial pathogens, yielding a more active AMP and/or a more selective AMP. Substituting Trp with halogenated tryptophan analogues at the peptide’s position 1 demonstrated differential effects on antimicrobial efficacy, leading to strain-specific enhancements. The same or lower efficacy with methylated tryptophan variants further underscores the importance of carefully evaluating chemical modifications to maintain antimicrobial potency and selectivity. This work demonstrates the successful biosynthetic production of halogenated tryptophan analogue-containing nisin variants and this offers a promising avenue for the engineering of lantibiotics to effectively and specifically target pathogens.

## Supplementary Material



## Data Availability

All data supporting the findings of this study are available within the paper and its Supporting Information files.

## Notes

Strains and plasmids used in this study; molecular masses and the incorporation efficiency of precursor peptides of nisin mutant I1W with Trp or various tryptophan analogues introduced at position 1; MS/MS fragmentation analysis of the core peptides (−8H_2_O) of 5FW-I1W, 5BW-I1W and 5MW-I1W purified after NisP cleavage and HPLC purification; comparison of HPLC spectra (226 nm) for the core peptides of I1W and its variants (PDF)
