# Supplementary material for: Synthesis and Antimicrobial Specificities of Halogenated Tryptophan-Containing Nisin Variants
Source: ACS Chem Biol. 2025 Sep 12;20(10):2503–11. doi: 10.1021/acschembio.5c00632 (PMC12538548; doi:10.1021/acschembio.5c00632)
Supplement: Supplementary file 1 [file cb5c00632_si_001.pdf]

SUPPLEMENTARY INFORMATION

Synthesis and Antimicrobial Specificities of Halogenated Tryptophan-Containing Nisin Variants

Chenhui Wang[a], Sanne Tervoort[a], Oscar P. Kuipers[a], Jaap Broos[a*]

[a]Department of Molecular Genetics, Groningen Biomolecular Sciences and Biotechnology Institute, University of Groningen, Groningen, the Netherlands

[*]Correspondence to Jaap Broos

E-mail address: j.broos@rug.nl

Table S1. Strains and plasmids used in this study

Strains or plasmids	Characteristics	Source
**Strains**		
Lactococcus lactis PA1002	*L. lactis* Trp auxotroph, peptide expression	[1]
Bacillus cereus CH-85	Indicator strain	MOLGEN Lab collection
Staphylococcus aureus LMG15975 (MRSA)	Indicator strain	MOLGEN Lab collection
Staphylococcus aureus LMG10147	Indicator strain	MOLGEN Lab collection
Enterococcus faecium LMG16003 (VRE)	Indicator strain	MOLGEN Lab collection
**Plasmids**		
pNZ-nisin(I1W)-TrpRS	Nisin mutation (I1W) with the overexpression TrpRS, P_{nisA} promoter, Cm^R	[2]
pTLReBTC	*nisBTC*, encoding nisin modification machinery, P_{czcD} promoter, Ery^R	[2]

Table S2. Molecular masses and the incorporation efficiency of precursor peptides of nisin mutant I1W with Trp or various tryptophan analogues introduced at position 1

Peptides (I1W nisin)	Modification	Predicted mass (Da)		Measured mass (Da)		Incorporation efficiency
		+Met1	-Met1	+Met1	-Met1	
I1W	$-8H_2O$	5892.03	5760.82		5760.29	
	$-7H_2O$	5910.05	5778.84		5778.26	
	$-6H_2O$	5928.07	5796.86		5801.33	
5FW-I1W	$-8H_2O$	5910.02	5778.81		5778.26	98%
	$-7H_2O$	5928.04	5796.83		5796.24	
	$-6H_2O$	5946.06	5814.85		5819.35	
5CW-I1W	$-8H_2O$	5926.47	5795.26		5795.17	86%
	$-7H_2O$	5944.49	5813.28		5812.17	
	$-6H_2O$	5962.51	5831.30		5827.23	
5BW-I1W	$-8H_2O$	5970.92	5839.71		5839.07	86%
	$-7H_2O$	5988.94	5857.73		5857.07	
	$-6H_2O$	6006.96	5875.75		5874.13	
5MW-I1W	$-8H_2O$	5906.05	5774.84		5774.22	91%
	$-7H_2O$	5924.07	5792.86		5792.21	
	$-6H_2O$	5942.09	5810.88		5808.20	

A

MS/MS fragmentation of 5FW-I1W (-8H_2O) core peptide

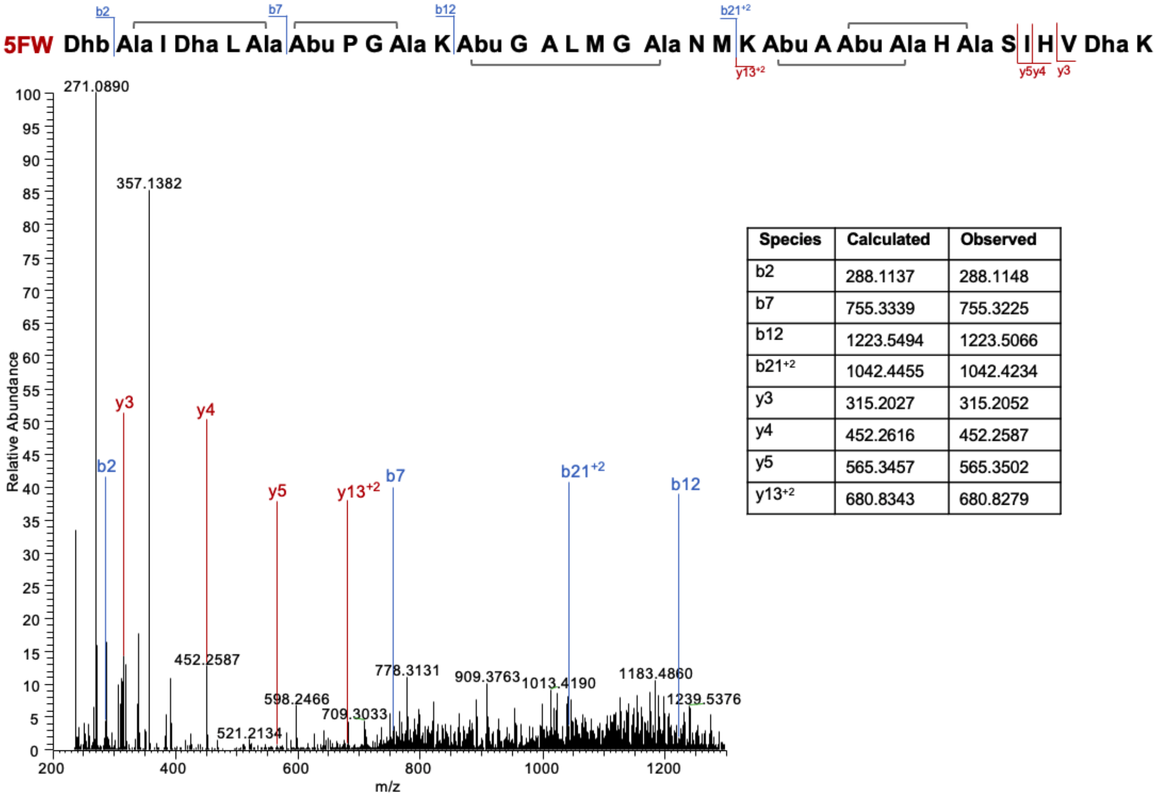

Species	Calculated	Observed
b2	288.1137	288.1148
b7	755.3339	755.3225
b12	1223.5494	1223.5066
$b21^{+2}$	1042.4455	1042.4234
y3	315.2027	315.2052
y4	452.2616	452.2587
y5	565.3457	565.3502
$y13^{+2}$	680.8343	680.8279

B

MS/MS fragmentation of 5BW-I1W (-8H_2O) core peptide

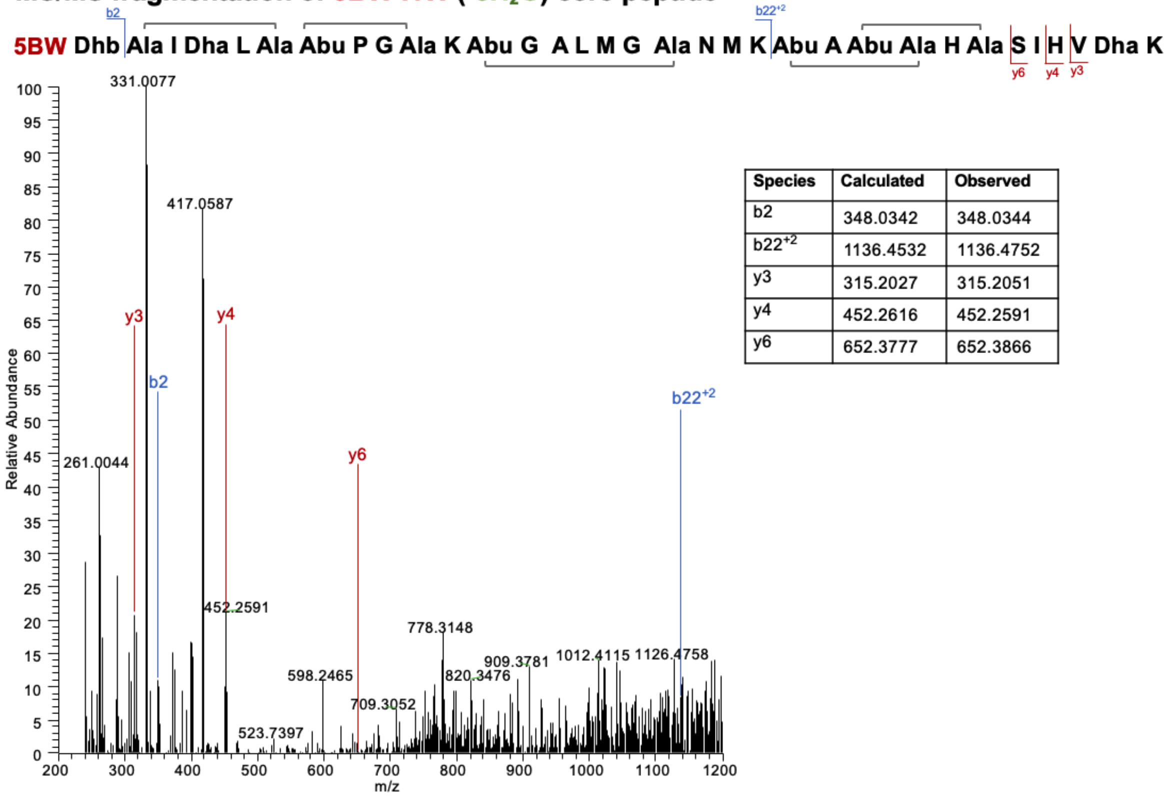

Species	Calculated	Observed
b2	348.0342	348.0344
$b22^{+2}$	1136.4532	1136.4752
y3	315.2027	315.2051
y4	452.2616	452.2591
y6	652.3777	652.3866

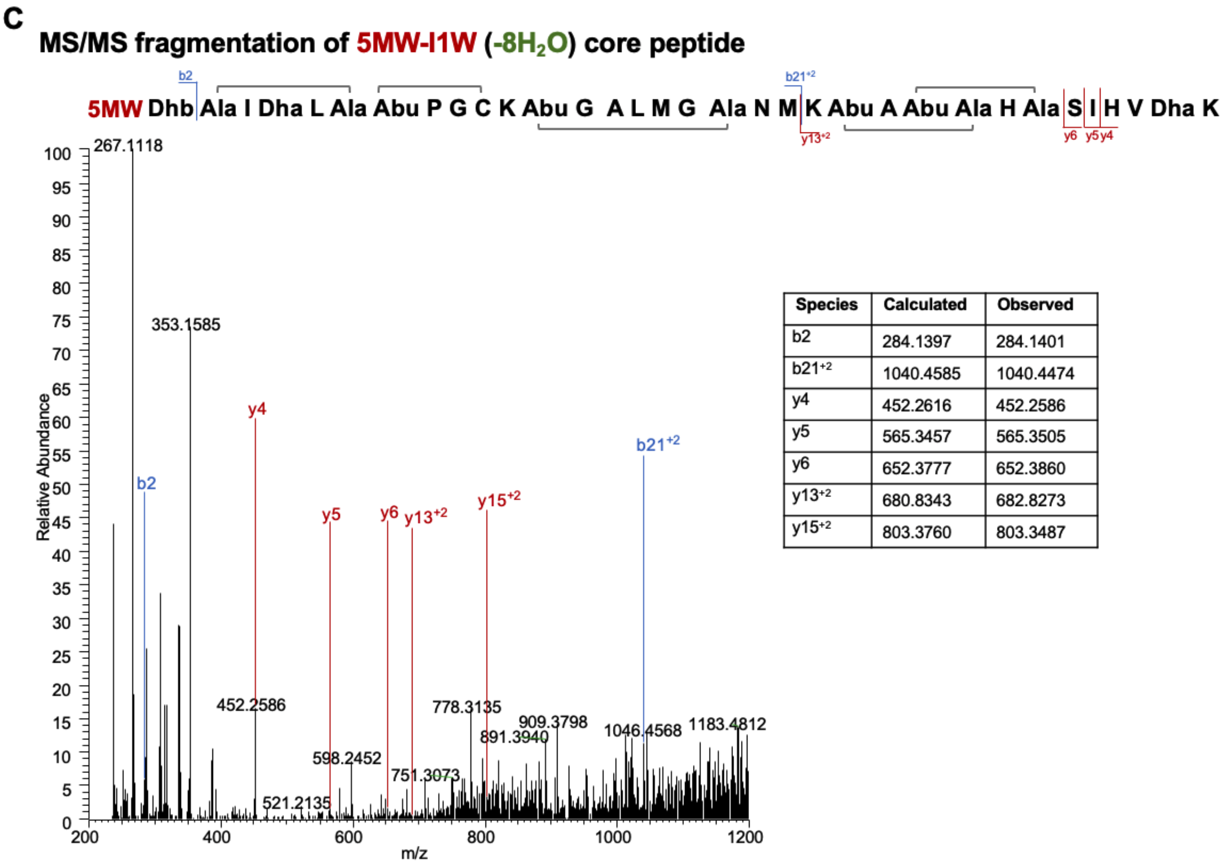

Species	Calculated	Observed
b2	284.1397	284.1401
b21+2	1040.4585	1040.4474
y4	452.2616	452.2586
y5	565.3457	565.3505
y6	652.3777	652.3860
y13+2	680.8343	682.8273
y15+2	803.3760	803.3487

Figure S1. MS/MS fragmentation analysis of the core peptides (-8H_2O) of I1W and its variants purified after NisP cleavage and HPLC purification. (A) 5FW-I1W; (B) 5BW-I1W; (C) 5MW-I1W.

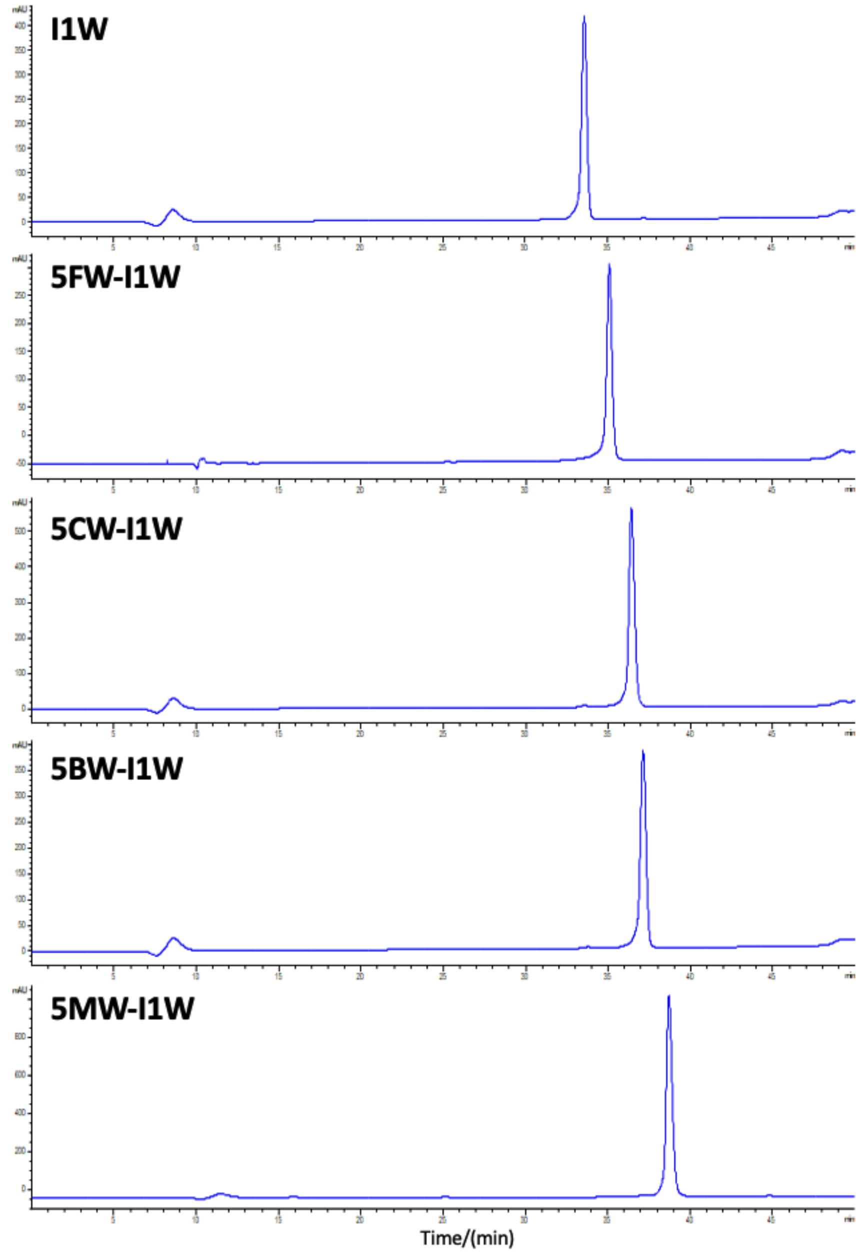

Figure S2. HPLC chromatograms (226 nm) of the purified core peptides I1W and its variants.

References

(1) El Khattabi, M.; van Roosmalen, Maarten L.; Jager, D.; Metselaar, H.; Permentier, H.; Leenhouts, K.; Broos, J. Lactococcus lactis as expression host for the biosynthetic incorporation of tryptophan analogues into recombinant proteins. *Biochemical Journal* **2007**, *409* (1), 193-198.

(2) Guo, L.; Kuipers, O. P.; Broos, J. Facile Halogenation of Antimicrobial Peptides As Demonstrated by Producing Bromotryptophan-Labeled Nisin Variants with Enhanced Antimicrobial Activity. *J. Nat. Prod.* **2024**, *87* (6), 1548-1555.
